# Effects of community-based open and closed skill exercise interventions on ecological executive function and mobile phone dependence of adolescents

**DOI:** 10.3389/fpsyg.2025.1694637

**Published:** 2025-12-11

**Authors:** Chao Xie, Ziyun Zhang, Shuai Wang, Jun Zhang, Yu Jin

**Affiliations:** 1 School of Physical Education, Hubei Minzu University, Enshi, China; 2 School of Life and Health,Huzhou College, Huzhou, China; 3 Library, Hubei Minzu University, Enshi, China; 4 School of Physical Education, Liaoning Normal University, Dalian, China; 5 School of Physical Education, Hubei University of Arts and Science, Xiangyang, China

**Keywords:** ecological executive function, mobile phone dependence, open skill, closed skill, physical exercise

## Abstract

**Objective:**

Taking the community as the intervention setting, this study aims to explore the intervention effects of community-based open-skill and closed-skill exercises on adolescents' ecological executive function and mobile phone dependence. It further verifies the mediating role of ecological executive function in the relationship between exercise types and mobile phone dependence, so as to provide scientific basis for communities to formulate precise intervention programs for adolescents' mobile phone dependence.

**Methods:**

A randomized controlled trial design was adopted. A total of 61 adolescents with mobile phone dependence were recruited (aged 13–15 years; the open-skill exercise group had a mean age of 13.76 ± 0.45 years, and the closed-skill exercise group had a mean age of 13.57 ± 0.79 years). They were randomly divided into the open-skill exercise group (31 participants, including 24 boys accounting for 77.42% and 7 girls accounting for 22.58%) and the closed-skill exercise group (30 participants, including 18 boys accounting for 60.00% and 12 girls accounting for 40.00%). The intervention was of moderate intensity, with duration of 8 weeks, 3 sessions per week, and 60 min per session. The Adolescent Executive Function Scale and Adolescent Mobile Phone Dependence Self-Rating Questionnaire were used to assess the adolescents' ecological executive function and the degree of mobile phone dependence. Statistical analyses, including independent samples *t*-test, chi-square test, multivariate analysis of variance, Pearson correlation analysis, and mediating effect test, were conducted using SPSS 21.0 and AMOS 16.0 software.

**Results:**

Open-skill exercise was more beneficial for improving adolescents' ecological inhibitory control (η^2^ = 0.103) and ecological working memory (η^2^ = 0.200; *p* < 0.05). It was also more effective in reducing adolescents' mobile phone dependence (η^2^ = 0.290), as well as withdrawal symptoms (η^2^ = 0.105), craving (η^2^ = 0.145), and physical and psychological impacts (η^2^ = 0.296) caused by mobile phone dependence (*p* < 0.05). Further mediating effect test showed that open-skill exercise could indirectly reduce mobile phone dependence by improving ecological inhibitory control and ecological working memory.

**Conclusion:**

Community-based open-skill exercise is significantly superior to closed- skill exercise in improving adolescents' ecological executive function and reducing mobile phone dependence. Moreover, ecological inhibitory control and ecological working memory play a mediating role between open-skill exercise and mobile phone dependence. The results of this study provide scientific support for communities to formulate precise intervention programs for adolescents' mobile phone dependence, and popularizing open-skill exercise can help alleviate the problem of excessive mobile phone dependence among adolescents.

## Introduction

With the rapid development of mobile Internet technology and the popularization of smartphones, mobile phones have become an indispensable tool in adolescents' daily lives (Papale et al., 2025). However, the phenomenon of excessive dependence has subsequently emerged as a global public health issue (Peraman and Parasuraman, 2016). Mobile phone dependence refers to a state of behavioral addiction caused by individuals' excessive use of mobile phones, which is characterized by a strong craving for mobile phone use, inability to control the frequency and duration of use, neglect of responsibilities and social interactions in real life due to phone use, and even the occurrence of adverse physical or psychological consequences (De-Sola Gutiérrez et al., 2016; Park, 2005). Globally, the prevalence of adolescent mobile phone dependence is showing a significant upward trend. A systematic review (Sahu et al., 2019), which included 12 original studies and provided a global overview of the prevalence of mobile phone dependence among children and adolescents, found that the problematic mobile phone usage rate was 6.3% in the total population, with 6.1% among boys and 6.5% among girls. As the world's largest market for mobile phone users, China also cannot afford to ignore the issue of adolescent mobile phone dependence. A study (Wang et al., 2022) on adolescents in Yunnan Province, China found that the detection rate of mobile phone dependence among adolescents in this region reached 11.11%. In addition, a survey by Wang et al. (2025) showed that the overall prevalence of mobile phone dependence among middle school students in Guangzhou, China, was 10.0% in 2023, with 7.3% among junior high school students and 12.4% among senior high school students. Therefore, there is an urgent need for scientific and effective intervention methods to reduce adolescents' mobile phone dependence.

Physical exercise, as a low-cost and easily promotable intervention method, has been proven in recent years to have a positive improving effect on mobile phone dependence (Shi et al., 2025; Yang et al., 2022). At present, relevant research mainly focuses on exploring the dose-effect of exercise intervention, i.e., investigating the optimal duration, intensity, and frequency of exercise. A meta-analysis by Li et al. (2023) found that exercise intervention can significantly reduce mobile phone dependence among adolescents, and exercise lasting more than 8 weeks, at least 3 times per week, and 30–60 min per session is more conducive to reducing mobile phone dependence. A meta-analysis by Chen et al. (2025) revealed that exercise could alleviate mobile phone dependence in children and adolescents. Specifically, aerobic exercise with duration of 12 weeks, moderate intensity, 3 times a week, and 40–45 min each time was more likely to achieve the expected intervention effect of improving mobile phone dependence in children and adolescents. However, current research has paid insufficient attention to exercise types, making it difficult to provide more precise guidance for exercise practice.

There is a close association between executive function and mobile phone dependence (Tauste-Garcia et al., 2025). Individuals with stronger executive function exhibit lower levels of mobile phone dependence (Li et al., 2025). Executive function refers to the advanced psychological ability of individuals to supervise, regulate, and coordinate their own cognitive processes when completing complex cognitive tasks. It mainly includes three core components: inhibitory control, working memory, and cognitive flexibility (Baggetta and Alexander, 2016). Low executive function is an important risk factor for mobile phone dependence among adolescents (Oh et al., 2021; Reed, 2023). Adolescents with weak inhibitory control find it harder to resist the temptation of information pushed by mobile phones, and individuals with insufficient cognitive flexibility are more likely to fall into the vicious cycle of “mobile phone use—avoidance of reality” (Oh et al., 2021; Reed, 2023). Therefore, exploring intervention measures that can effectively promote executive function may reduce adolescents' mobile phone dependence.

Physical exercise has been proven to be an effective means of improving executive function (Singh et al., 2025). A large number of studies (Berchicci et al., 2013; Moore et al., 2022; Seifert et al., 2010) have shown that regular physical exercise can promote the development of executive function by increasing the level of brain-derived neurotrophic factor (BDNF) and enhancing the neural connections between the prefrontal cortex and the hippocampus. Notably, existing studies (Shi and Feng, 2022; Shi et al., 2022; Hu et al., 2025) have classified exercise types into two categories: open-skill and closed-skill exercise, and explored their intervention effects on adolescents' executive function, finding that open-skill exercise yields better intervention effects than closed-skill exercise. This may be because open-skill exercise requires continuous processing of multi-source information in a dynamic environment, which places higher demands on the core components of executive function, thereby producing a stronger “cognitive training” effect (Cheng et al., 2023).

However, existing studies have obvious limitations. Firstly, the effects of different exercise types (open and closed motor skills) on intervening in adolescent mobile phone dependence remain unclear. Secondly, in executive function assessments, most studies adopt traditional laboratory computer-based tasks (e.g., the Stroop task, N-back task) to evaluate executive function. Although such tasks can accurately measure specific cognitive components, they are disconnected from daily life scenarios and thus difficult to reflect individuals' functional performance in real environments. As an assessment perspective emphasizing adaptation to real-life contexts, ecological executive function focuses more on individuals' ability to apply executive function to solve practical problems in daily tasks (Gioia et al., 2010). However, its association with exercise types has not been fully explored. Finally, although studies have confirmed the correlation between executive function and mobile phone dependence, the mediating effect of executive function between exercise intervention and mobile phone dependence remains unclear, and detailed data support is urgently needed.

As an important setting for adolescents' lives, communities have inherent advantages in carrying out group interventions. Based on this, this study aims to explore the intervention effects of community-based open-skill and closed-skill exercises on adolescents' ecological executive function and mobile phone dependence. It intends to compare the intervention effects of the two types of skill exercises on ecological executive function and reducing mobile phone dependence, and further investigate the potential mediating role of ecological executive function in the relationship between exercise types and mobile phone dependence. Through this study, it is expected to enrich the research perspective in the field of exercise and cognitive development, clarify the differential effects of open-skill and closed-skill exercises, and provide new evidence for the association mechanism of “exercise type—cognitive function—behavioral addiction”. In addition, this study will provide a scientific basis for communities to design precise health intervention programs for adolescents, helping to solve the social problem of adolescent mobile phone dependence.

## Methods

### Participants

Based on the results of a systematic review and meta-analysis by Feng et al. (2023), this study extracted the effect size (*ES* = 0.803) of open skill exercise interventions on the executive function of children and adolescents. Using G • Power 3.1 software, calculations were performed based on the difference test for comparing means between two groups, with the significance level set at α = 0.05 and the test power at 1—β = 0.80. The ratio of sample sizes between the two groups was defined as 1:1, and the total number of participants required was calculated to be 40. In addition, considering the potential invalidity of participant data, this study calculated that 48 adolescents aged 13–15 need to be recruited based on an expected invalid data rate of 20%, including 24 adolescents in the open-skill exercise group and 24 in the closed-skill exercise group.

Participants voluntarily participating in this intervention study were recruited in Enshi City, Hubei Province, China. A total of 82 participants were recruited, all of whom were required to have normal intelligence and no cognitive or mental disorders. This study used the Self-Rating Questionnaire for Adolescent Mobile Phone Dependence developed by Tao et al. (2025) for preliminary screening, where a score of ≥ 27 was regarded as mobile phone dependence. Based on this, 21 adolescents without mobile phone dependence were excluded, leaving 61 adolescents to participate in the intervention study. They were subsequently divided into the open skill exercise group and the closed skill exercise group, with 31 participants in the open skill exercise group and 30 in the closed skill exercise group. This study was conducted in compliance with the Declaration of Helsinki and was approved by the Scientific Research Ethics Committee of Hubei Minzu University. Moreover, all participants and their guardians expressed their voluntary participation in the intervention experiment.

Descriptive statistics were performed on the basic information of the two groups, and the details are shown in Table 1. The mobile phone dependence of the open skill exercise group was (56.45 ± 5.02), and that of the closed motor skill exercise group was (56.27 ± 4.99), so both groups had relatively severe mobile phone dependence; the age of participants in the open skill exercise group was (13.76 ± 0.45) years, while that in the closed motor skill exercise group was (13.57 ± 0.79) years, with no significant difference between the two groups (*t* = 1.167, *p* = 0.249); the proportion of girls in the open skill exercise group was 22.58%, and that in the closed motor skill exercise group was 40.00%, with no significant difference between the two groups (χ^2^ = 1.421, *p* = 0.233); regarding parental education level, 51.61% of participants in the open skill exercise group had fathers with a junior high school education, compared with 53.33% in the closed motor skill exercise group, and 38.71% of participants in the open skill exercise group had mothers with a junior high school education, vs 46.67% in the closed motor skill exercise group, with no significant differences between the groups (*p* > 0.05). Additionally, there were no statistically significant differences between the two groups in terms of ecological executive function (inhibitory control, working memory, and cognitive flexibility) and mobile phone dependence-related indicators (withdrawal symptoms, craving, and physical and mental impacts) (all *p* > 0.05). Moreover, no statistically significant differences were observed between the two groups in physical activity level, Body Mass Index (BMI), and Family Socioeconomic Status (FSES) (all *p* > 0.05).

**Table 1:** Basic information of participants.

**Variables**	**OS**	**CS**	***t/χ^2^***	***p***
Age	13.76 ± 0.45	13.57 ± 0.79	1.167	0.249
**Gender** ^*^			1.421	0.233
Boys	24 (77.42%)	18 (60.00%)		
Girls	7 (22.58%)	12 (40.00%)		
**Father's edu**.^*^			5.823	0.054
Primary school and below	4 (12.90%)	10 (33.33%)		
Junior school	16 (51.61%)	16 (53.33%)		
Senior school and above	11 (35.48%)	4 (13.33%)		
**Mother's edu**.^*^			4.205	0.122
Primary school and below	8 (25.81%)	12 (40.00%)		
Junior school	12 (38.71%)	14 (46.67%)		
Senior school and above	11 (35.48%)	4 (13.33%)		
Inhibitory control	11.84 ± 3.91	12.07 ± 2.79	−0.263	0.794
Working memory	12.87 ± 4.73	12.80 ± 2.91	0.071	0.944
Cognitive flexibility	14.23 ± 5.63	14.40 ± 1.47	−0.164	0.869
Withdrawal symptoms	26.16 ± 3.79	25.60 ± 3.77	0.580	0.564
Craving	12.65 ± 1.54	12.27 ± 1.95	0.844	0.402
Physical and mental impacts	17.65 ± 1.68	18.40 ± 1.65	−1.766	0.083
Mobile phone dependence	56.45 ± 5.02	56.27 ± 4.99	0.144	0.886
Physical activity level	43.83 ± 34.50	41.71 ± 27.28	0.237	0.814
BMI	22.26 ± 6.20	24.78 ± 6.10	−1.594	0.116
FSES	3.00 ± 0.00	3.00 ± 0.00	NA	NA

^*^ indicates that the chi-square test was used for statistical analysis; OS represents the open skill exercise group; CS represents the closed skill exercise group; edu. stands for education level; BMI stands for Body Mass Index; FSES stands for Family Socioeconomic Status; NA means not applicable.

### Study design

This study adopts a randomized controlled trial design. For allocation concealment, a central randomization system is used in this study. An independent person who is not involved in recruitment and intervention implementation generates a random allocation sequence through a computer, and stores the sequence in sealed, opaque envelopes, with each envelope corresponding to a participant number. After eligible participants complete the baseline assessment, specialized recruiters open the envelopes in sequence and assign the participants to either the open-skill exercise group or the closed-skill exercise group according to the allocation results inside the envelopes. Among them, there are 31 participants in the open-skill exercise group and 30 participants in the closed-skill exercise group. In addition, due to the significant differences in intervention forms between open-skill exercises and closed-skill exercises in this study, it is difficult to completely blind the participants and the personnel implementing the intervention. Therefore, the single-blind method of outcome assessors is mainly adopted to ensure the accuracy of the assessment results.

This study uses a pre-test—post-test control group design. Before the intervention, the ecological executive function and mobile phone dependence of adolescents in both groups are assessed. During the intervention period, the open-skill exercise group carries out open-skill sports such as basketball and football; the closed-skill exercise group conducts closed-skill sports such as running and cycling. The specific duration, frequency, intensity, etc. of the intervention will be set with reference to the parameters proven effective in relevant studies to ensure the scientificity and effectiveness of the intervention. After the intervention, the ecological executive function and mobile phone dependence of adolescents are tested again.

### Study instruments

#### Ecological executive function

This study adopted the Adolescent Executive Function Scale (EFS-A) developed by Huang et al. (2014) to assess the ecological executive function of adolescents. The questionnaire consists of 21 items, covering three core dimensions of executive function: inhibitory control (6 items), working memory (7 items), and cognitive flexibility (8 items). The three dimensions of the questionnaire explain 45.39% of the variance, with factor loadings of each item ranging from 0.499 to 0.727, indicating that the questionnaire has ideal construct validity. In addition, the Cronbach's α coefficients of the scale and each factor range from 0.786 to 0.897, among which the coefficient of the total scale is higher than 0.8, and the α coefficients of each dimension are all above 0.7, fully proving that the questionnaire has good internal consistency reliability (Huang et al., 2014). The questionnaire uses a 3-point scoring method, where “1” represents “never”, “2” represents “sometimes”, and “3” represents “often”. Drawing on the research of Wang (2023), this study calculated the total score after reverse scoring of the questionnaire, that is, the higher the total score, the better the executive function of adolescents. The EFS-A can effectively address the limitations of traditional laboratory computer-based tasks and align with the core requirements of ecological executive function. First, the core limitation of traditional laboratory tasks lies in their disconnection from daily life. They only focus on the accurate measurement of single cognitive components and cannot reflect individuals' functional performance in complex real-world scenarios. In contrast, the EFS-A is designed entirely around the daily scenarios of adolescents. Second, ecological executive function emphasizes individuals' ability to apply executive function to solve practical problems in daily tasks, and the EFS-A precisely aligns with this goal.

#### Mobile phone dependence

This study used the Self-Rating Questionnaire for Adolescent Mobile Phone Dependence developed by Tao et al. (2025) to assess participants' mobile phone dependence. The questionnaire consists of 13 items, comprehensively outlining the manifestations of mobile phone dependence from three core dimensions: withdrawal symptoms (6 items), craving (3 items), and physical and mental impact (4 items). In terms of scoring, a Likert 5-point self-rating scale was adopted, with options scored from 1 to 5, corresponding to “completely inconsistent” to “completely consistent”. The minimum score of the questionnaire is 13, and the maximum score is 65, with higher scores indicating more severe mobile phone dependence. The Cronbach's α coefficient of the total scale reaches 0.87, and the Cronbach's α coefficient of each dimension range from 0.58 to 0.83, indicating that the questionnaire has good overall internal consistency (Tao et al., 2025).

#### Demographic information

Referring to previous studies (Houwen et al., 2017; Jirout et al., 2019), this study investigated participants' age, gender, height, weight, parental education level, and family economic status to analyze potential confounding factors. In this study, participants' BMI was calculated based on height and weight, with the formula: BMI = weight (kg)/height (m)^2^. For parental education level, referring to previous studies (Zhong and Wang, 2020; Zhong et al., 2020), “no education received” and “primary school” were coded as 1, i.e., “primary school and below”; “junior school” was coded as 2; “secondary technical school/technical school”, “vocational high school”, and “regular high school” were coded as 3. Considering that the proportion of students' parents with “junior college” and “bachelor's degree” was relatively small, they were also coded as 3, i.e., “senior school and above”. This study used a 5-point Likert self-rating scale to investigate participants' FSES, where 1 to 5 respectively represent very difficult, difficult, average, well-off, and very well-off. This self-rating method for FSES has been widely used in subsequent studies (Angleitner, 1978; Fu et al., 2023; Yuan et al., 2023).

#### Heart rate monitoring

In this study, heart rate was used for quantitative monitoring of exercise intensity to provide data on the effectiveness of the intervention. One session per week was selected from both open-skill exercise and closed-skill exercise classes, and 15 participants were chosen to wear POLAR heart rate monitors during the intervention. The selection rules for participants wearing heart rate monitors were as follows: students gathered in 4 rows; those in rows 1 and 3 wore the monitors in the first session, and those in rows 2 and 4 wore them in the second session, with this pattern cycling in sequence. A total of 16 heart rate monitoring sessions were conducted (8 in the open-skill exercise group and 8 in the closed-skill exercise group). In addition, participants who dropped out during the testing process were excluded. Referring to previous studies (Chen et al., 2014; Wang et al., 2020), the intensity of the intervention was set at moderate intensity, i.e., 60–69% HRmax.

#### Physical activity level

To mitigate the potential impact of physical activity outside the intervention period on the study results, this study collected information on all participants' pre- intervention physical activity using the Physical Activity Rating Scale (PARS-3) before the intervention, ensuring that there were no statistically significant differences in baseline exercise levels between the two groups. Developed by Liang (1994), this scale consists of three dimensions: exercise intensity, exercise frequency, and duration of each exercise session, with each dimension divided into 5 levels. In terms of scoring rules, the 5 levels of exercise intensity and exercise frequency are scored from 1 to 5 points respectively; the 5 levels of exercise duration are scored from 0 to 4 points. The formula for calculating the total physical activity volume is: Exercise Volume = Intensity × Duration × Frequency, with the final score ranging from 0 to 100 points. Referring to the research results of Liang (1994), physical exercise levels can be divided into three grades based on the exercise volume score: a score of 19 or below indicates low exercise volume, 20–42 points indicate moderate exercise volume, and 43 points or above indicate high exercise volume. This scale has been verified to have good reliability and validity (Liang, 1994) and is currently widely used in surveys on physical exercise among adolescent populations (Liang, 1994; Yang et al., 2021; Zhao, 2021).

### Intervention program

Drawing on previous studies (Feng et al., 2023; Shi et al., 2022), this study set the intervention period at 8 weeks, with a frequency of 3 times per week, and each exercise session lasting 60 min, including 10 min of warm-up, 40 min of formal exercise, and 10 min of cool-down. The open-skill exercise group engaged in open-skill sports such as basketball and football. During the formal exercise phase, various confrontational and collaborative exercises were arranged, such as team basketball games and football tactical coordination training, allowing adolescents to continuously make decisions and responses in a dynamically changing environment. The closed-skill exercise group performed closed-skill sports like running and cycling, with the same time allocation for warm-up, formal exercise, and cool-down as the open-skill exercise group. Running and cycling exercises could be set with different speed and distance requirements, and the movement process was relatively stable and predictable. The specific intervention contents for the open skill exercise group and the closed skill exercise group are detailed in Table 2.

**Table 2:** The specific intervention contents for the open skill exercise group and the closed skill exercise group.

**Weeks**	**Open skill exercise group**		**Closed skill exercise group**	
	**Stage**	**Main content**	**Stage**	**Main content**
Weeks 1–2	Basic adaptation stage	Basketball: dribbling around cones + passing drills; Football: basic dribbling + short-distance passing and receiving	Basic adaptation stage	Running: Interval walking-jogging, alternating 3 min of running and 2 minutes of walking in cycles; Cycling: Steady-speed cycling, with the speed controlled at 15–18 km/h.
Weeks 3–5	Skill improvement stage	Basic skills + simple confrontation: Basketball (3v3 small-court offense and defense + shooting training); Football (2v2 partial coordination + shooting practice). Add 1–2 new training modules each week (e.g., basketball pick-and-roll coordination, football defensive positioning) to ensure the content progresses in a step-by-step manner.	Intensity improvement stage	Moderate-intensity interval training: Running (alternating 5 min of steady running and 1 min of accelerated running in cycles, with heart rate maintained at 60–65% HRmax during the steady phase); Cycling (alternating 8 min of steady cycling and 2 min of cycling with a 5° incline increase in cycles).
Weeks 6–8	Comprehensive application stage	Full-scenario confrontation: Basketball (5v5 full-court game with simplified rules); Football (7v7 small-sided game), to enhance decision-making ability in dynamic environments.	Endurance maintenance stage	Continuous moderate-intensity exercise: Running (continuous steady running for 40 min, with speed adjusted according to individual HRmax); Cycling (continuous steady cycling for 40 min, with incline stabilized at 3–5°).

To ensure the effective implementation of the intervention program, this study first conducted unified training for the staff involved in the research. The training content included familiarizing themselves with the standard movements, teaching methods, and sports safety precautions of open and closed-skill exercise programs, as well as mastering communication skills with adolescents, so as to effectively guide and encourage adolescents to actively participate in the intervention process. During this process, the study developed separate manuals for the two exercise types, specifying the training modules, duration allocation, and movement standards for each weekly session. All intervention implementers were required to receive pre-training on the manuals and could only carry out the intervention after passing the assessment. Secondly, appropriate venues in the community were selected for the exercise activities. For example, community basketball courts and football fields were used for open-skill exercises, and fitness trails and bicycle lanes around the community were used for closed-skill exercises. Each exercise session was supervised on-site by professional coaches or trained staff to ensure sports safety and standardized movements. During the exercise, the intensity and difficulty of the movements were adjusted in a timely manner according to the actual situation of the adolescents to ensure that each adolescent could adapt to the exercise rhythm. At the same time, adolescents were encouraged to support and supervise each other to enhance their participation enthusiasm and persistence. In addition, during the intervention period of this study, participants were required to fill out the exercise record form daily to detail information about physical activities outside the intervention. Researchers verified and confirmed the forms weekly to ensure that participants' weekly physical activity remained at a basically consistent level. The weekly physical activity volume of participants is detailed in Supplementary Material 1.

### Mathematical statistics

In this study, SPSS 21.0 and AMOS 16.0 software was used for relevant data processing and statistical analysis. Firstly, the normality of the data was tested using the one-sample Shapiro-Wilk test combined with P-P plots and Q-Q plots. It was found that the data of relevant continuous variables approximately showed a normal distribution, so the independent samples *t*-test was used for inter-group comparative analysis. When conducting the independent samples *t*-test, the Levene test was used to test the homogeneity of variances. If the variances were homogeneous, the results assuming equal variances were used for analysis; if the variances were not homogeneous, the results not assuming equal variances were adopted. In addition, for categorical variables such as gender and parental education level, the chi-square test was used for inter-group comparative analysis. Secondly, this study adopted MANOVA to explore the effects of open and closed skill exercise interventions, where the dependent variables were the main outcome indicators of the pre-test and post-test, and the independent variable was the type of exercise (open skill exercise and closed skill exercise). According to the research purpose, the main effects of exercise types and the inter-group effects between pre-test and post-test were mainly reported. In this study, the Levene test was used to test the homogeneity of variances; the Pillai's trace method was used to test the model effects; and the estimated marginal means (LSD method) were used for pairwise comparisons. Thirdly, a partial Pearson correlation analysis controlling for baseline data and relevant confounding factors was used to explore the correlation between ecological executive functions and mobile phone dependence. Finally, the mediating effect test was used to explore the potential mediating role of ecological executive function (with baseline levels and confounding factors controlled) in the relationship between exercise and mobile phone dependence. To test the significance of the mediating effects, we conducted bias-corrected bootstrap tests with a 95% confidence interval (*CI*) from 5,000 resamples. For data with incomplete information, including 3 items such as height and weight, and 4 items such as physical activity level, this study used linear interpolation for imputation. In this study, *p* < 0.05 was considered statistically significant for differences.

## Results

### Effects of open and closed skill exercise interventions on ecological executive function

**Figure 1:**
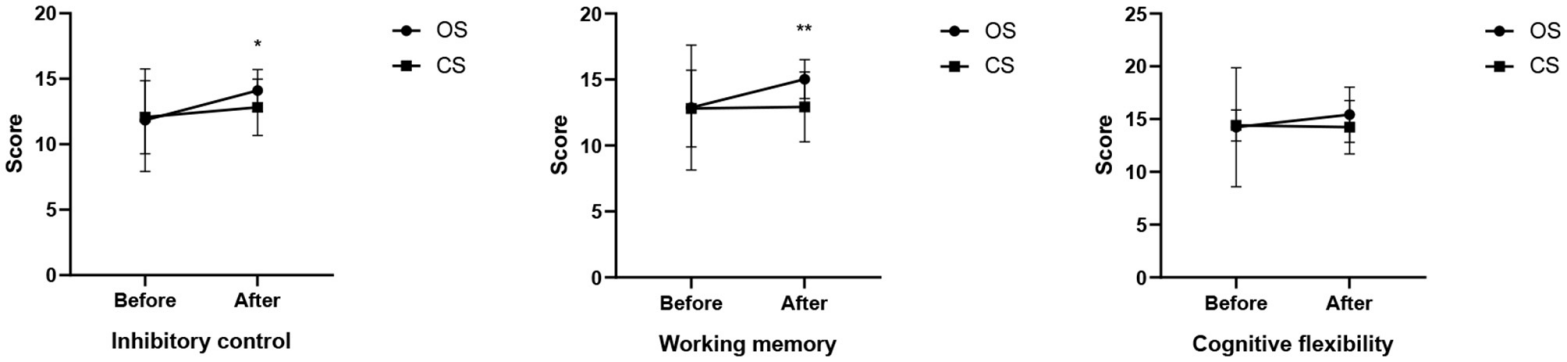
The changing trend of open and closed skill training interventions on adolescents' ecological executive function (*P < 0.05; **P < 0.01).

For ecological inhibitory control, the effect of exercise type was significant (*F* = 3.350, *p* = 0.042, η^2^ = 0.104), with the open skill exercise group showing significantly higher inhibitory control than the closed skill exercise group. Further tests revealed that the effect of the pre-test was not significant (*F* = 0.068, *p* = 0.795, η^2^ = 0.001), while the effect of the post-test was significant (*F* = 6.809, *p* = 0.011, η^2^ = 0.103). The score of the open skill exercise group (14.10 ± 1.60) was significantly higher than that of the closed skill exercise group (12.07 ± 2.79). For ecological working memory, the effect of exercise type was significant (*F* = 7.389, *p* = 0.001, η^2^ = 0.203), and the working memory of the open skill exercise group was significantly higher than that of the closed skill exercise group. Further tests indicated that the effect of the pre-test was not significant (*F* = 0.005, *p* = 0.944, η^2^ = 0.000), whereas the effect of the post-test was significant (*F* = 14.741, *p* = 0.000, η^2^ = 0.200). The score of the open skill exercise group (15.03 ± 1.47) was significantly higher than that of the closed skill exercise group (12.93 ± 2.65). For ecological cognitive flexibility, the effect of exercise type was significant (*F* = 2.438, *p* = 0.096, η^2^ = 0.078), but there was no significant difference in cognitive flexibility between the open skill exercise group and the closed skill exercise group. Further tests showed that neither the effect of the pre-test (*F* = 0.027, *p* = 0.870, η^2^ = 0.000) nor the effect of the post-test (*F* = 3.237, *p* = 0.077, η^2^ = 0.052) was significant. The changing trend of open and closed skill training interventions on adolescents' ecological executive function is detailed in Figure 1.

### Effects of open and closed skill exercise interventions on mobile phone dependence

**Figure 2:**
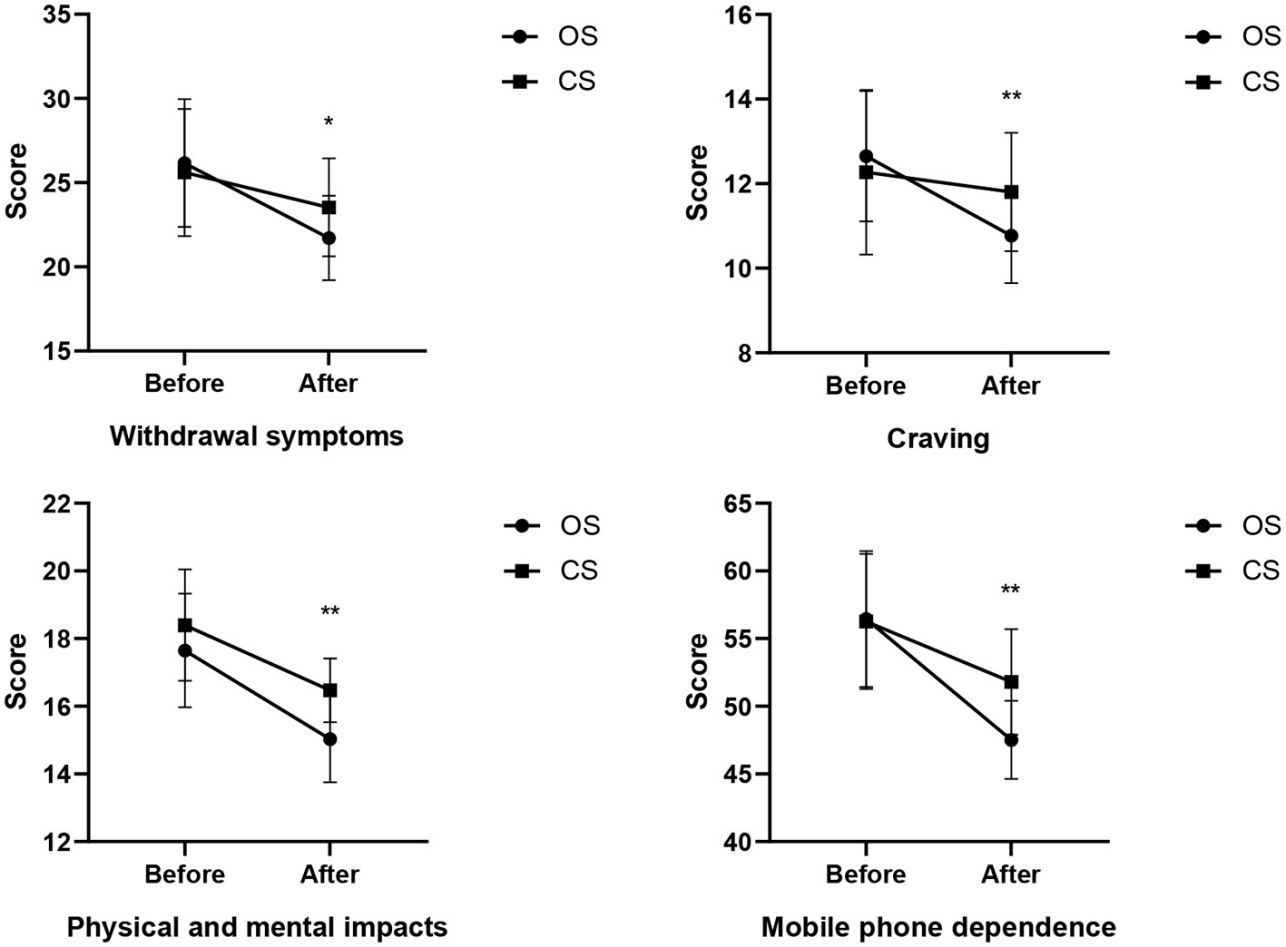
The changing trend of open and closed skill training interventions on adolescents' mobile phone dependence (*P < 0.05; **P < 0.01).

Regarding withdrawal symptoms, the effect of exercise type was significant (*F* = 5.641, *p* = 0.006, η^2^ = 0.163), with the withdrawal symptoms in the open-skill exercise group being significantly lower than those in the closed-skill exercise group. Further tests revealed that the pre-test effect was not significant (*F* = 0.337, *p* = 0.564, η^2^=0.006), while the post-test effect was significant (*F* = 6.895, *p* = 0.011, η^2^ = 0.105). Specifically, the score of the open-skill exercise group (21.71 ± 2.51) was significantly lower than that of the closed-skill exercise group (23.53 ± 2.91). In terms of craving, the effect of exercise type was significant (*F* = 7.961, *p* = 0.000, η^2^ = 0.001), and the craving level of the open-skill exercise group was significantly lower than that of the closed-skill exercise group. Further tests showed that the pre-test effect was not significant (*F* = 0.712, *p* = 0.402, η^2^ = 0.012), whereas the post-test effect was significant (*F* = 10.046, *p* = 0.002, η^2^ = 0.145). The score of the open-skill exercise group (10.77 ± 1.12) was significantly lower than that of the closed-skill exercise group (11.80 ± 1.40). For physical and mental impacts, the effect of exercise type was significant (*F* = 12.928, *p* = 0.000, η^2^ = 0.308), with the physical and mental impacts on the open-skill exercise group being significantly lower than those on the closed-skill exercise group. Further tests indicated that the pre-test effect was not significant (*F* = 3.119, *p* = 0.083, η^2^ = 0.050), but the post-test effect was significant (*F* = 24.864, *p* = 0.000, η^2^ = 0.296). The score of the open-skill exercise group (15.03 ± 1.28) was significantly lower than that of the closed-skill exercise group (16.47 ± 0.94). With respect to mobile phone dependence, the effect of exercise type was significant (*F* = 16.824, *p* = 0.000, η^2^ = 0.367), and the mobile phone dependence of the open-skill exercise group was significantly lower than that of the closed-skill exercise group. Further tests demonstrated that the pre-test effect was not significant (*F* = 0.021, *p* = 0.886, η^2^ = 0.000), while the post-test effect was significant (*F* = 24.044, *p* = 0.000, η^2^ = 0.290). The score of the open-skill exercise group (47.52 ± 2.89) was significantly lower than that of the closed-skill exercise group (51.80 ± 3.89). The changing trends of open-skill and closed-skill exercises in intervening in adolescents' mobile phone dependence are detailed in Figure 2.

### Correlation between ecological executive function and mobile phone dependence

After controlling for relevant confounding factors and baseline data, this study further conducted a Pearson correlation analysis between ecological executive function and mobile phone dependence among adolescents post-intervention. The results (Table 3) showed that inhibitory control was significantly (*p* < 0.01) negatively correlated with withdrawal symptoms (*r* = −0.417) and mobile phone dependence (*r* = −0.402); working memory was significantly (*p* < 0.05) negatively correlated with withdrawal symptoms (*r* = −0.476), craving (*r* = −0.286), and mobile phone dependence (*r* = −0.496). In addition, the correlation between cognitive flexibility and mobile phone dependence as well as its various dimensions was not significant (*p* > 0.05).

**Table 3:** Results of the partial Pearson correlation analysis between ecological executive function and mobile phone dependence after intervention.

**Variables**	**Withdrawal symptoms**	**Craving**	**Physical and mental impacts**	**Mobile phone dependence**
Inhibitory control	−0.417^**^	−0.066	−0.243	−0.402^**^
Working memory	−0.476^**^	−0.286^*^	−0.195	−0.496^**^
Cognitive flexibility	0.095	−0.143	−0.172	−0.030

^*^P < 0.05; ^**^P < 0.01; the confounding factors included age, gender, BMI, FSES, and parental education level.

### Test of the mediating role of ecological executive function between open skill exercise and mobile phone dependence

**Figure 3:**
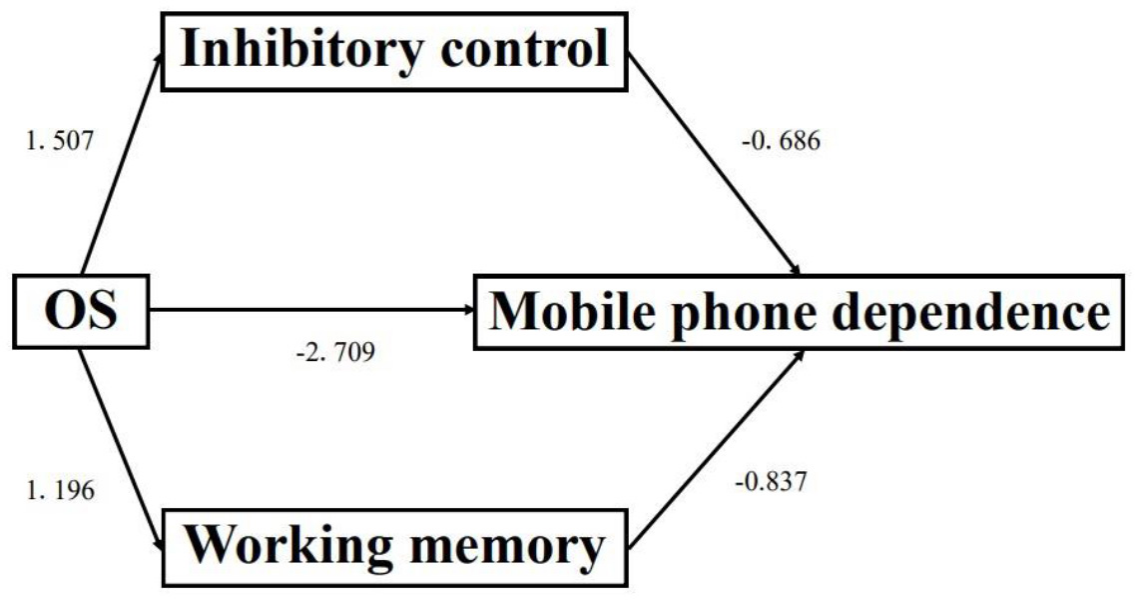
Path analysis diagram.

Based on the correlation analysis, this study further explored the potential mediating effect of ecological executive function between open-skill exercise and mobile phone dependence. The results of the model fit test showed that χ^2^/*df* = 1.568, GFI = 0.944, AGFI = 0.918, RMSEA = 0.035, NFI = 0.974, TLI = 0.914, and CFI = 0.969; therefore, the path model had a good fit. The results of the path analysis test indicated that: open-skill exercise had significant effects on inhibitory control (*ES* = 1.507, 95%*CI* = 0.670 ~ 2.344, *p* < 0.001), working memory (*ES* = 1.196, 95%*CI* = 0.263 ~ 2.219, *p* = 0.012), and cognitive flexibility (*ES* = 3.094, 95%*CI* = 2.195 ~ 3.993, *p* < 0.001); open-skill exercise exerted a significant effect on mobile phone dependence (*ES* = −2.709, 95%*CI* = −1.016 ~ −4.402, *p* = 0.002); both inhibitory control (*ES* = −0.686, 95%*CI* = −1.043 ~ −0.329, *p* < 0.001) and working memory (*ES* = −0.837, 95%*CI* = −1.156 ~ −0.518, *p* < 0.001) had significant effects on mobile phone dependence. However, the effect of cognitive flexibility on smartphone dependence was not significant (*p* > 0.05). In summary, the direct effect of open-skill exercise on mobile phone dependence was −2.709; the indirect effect of open-skill exercise on mobile phone dependence through inhibitory control was 1.507 × −0.686 = −1.035; the indirect effect of open-skill exercise on mobile phone dependence through working memory was 1.196 × −0.837 = −0.991. The results of the path analysis are detailed in Table 4; for details of the path analysis diagram, see Figure 3.

**Table 4:** The results of path analysis (bootstrap).

**Num**.	**Path**	***ES***	**95%*CI***	***SE***	***CR***	***p***
1	Inhibitory control ← OS	1.507	(0.670, 2.344)	0.427	3.530	< 0.001
2	Working memory ← OS	1.196	(0.263, 2.129)	0476	2.511	0.012
3	Cognitive flexibility ← OS	3.094	(2.195, 3.993)	0.459	6.734	< 0.001
4	Mobile phone dependence ← OS	−2.709	(−1.016, −4.402)	0.864	−3.135	0.002
5	Mobile phone dependence ← Inhibitory control	−0.686	(−1.043, −0.329)	0.182	−3.775	< 0.001
6	Mobile phone dependence ← Working memory	−0.837	(−1.156, −0.518)	0.163	−5.138	< 0.001
7	Mobile phone dependence ← Cognitive flexibility	0.317	(−0.014, 0.648)	0.169	1.876	0.061

## Discussions

### Open-skill exercise has a more prominent intervention effect on the ecological executive function of adolescents

The results of this study showed that open-skill exercise had a more significant intervention effect on ecological executive function than closed-skill exercise, with this effect being particularly prominent in terms of ecological inhibitory control and ecological working memory. These findings support previous research results based on computer-based test tasks (Li et al., 2024; Yakushina et al., 2025). For example, Yakushina et al. (2025) found that football exercise is more effective than martial arts exercise in improving visuospatial working memory. The reason why ecological tests and computer-based tests yield similar results lies in the fact that although the two types of tests differ in form, they both accurately target the core cognitive processing mechanisms of executive function and meet the core criteria for executive function assessment (Naglieri and Otero, 2013; Suchy, 2009). Moreover, the intervention effect of open-skill exercise on these mechanisms exhibits cross-scenario consistency.

Open-skill exercise has a better intervention effect on ecological executive function than closed-skill exercise, especially in terms of ecological inhibitory control and ecological working memory. The core reason lies in the differences in cognitive demands between the two types of exercise. Among these, the dynamic adaptability of open-skill exercise is highly aligned with the core requirements of ecological executive function. Specifically, both require flexible cognitive regulation in real-life scenarios, thus activating and strengthening the target cognitive mechanisms more accurately and frequently (Cañas et al., 2003). The core of ecological inhibitory control is to proactively inhibit irrelevant impulses or incorrect responses in complex and dynamic real-life scenarios, so as to ensure the priority execution of target behaviors (van den Wildenberg et al., 2022). In open-skill exercise, such “inhibition demands” run through the entire process; this kind of real-time inhibition training in a dynamic environment is completely consistent with the core requirement of ecological inhibitory control to cope with interference in real scenarios, and can directly strengthen the brain's ability to regulate impulsive behaviors (Wang et al., 2013). In contrast, the process of closed-skill exercise is highly fixed, requiring almost no response to sudden interference and rarely needing to proactively inhibit incorrect impulses, so it is naturally difficult to effectively intervene in ecological inhibitory control.

The core of ecological working memory is to store key information in a short period of time in real scenarios and flexibly retrieve it according to changes in the environment, emphasizing the dynamic processing of information (Ericsson and Delaney, 1999). Open-skill exercise exactly imposes high-intensity demands on this dynamic memory ability; this training mode of “storing, adjusting, and retrieving information simultaneously” is highly consistent with the core requirement of ecological working memory to “cope with information changes in real scenarios”, and can directly train the brain's ability to manage and manipulate short-term information (Seidler et al., 2012; Wang, 2016). The information demands of closed-skill exercise are single and fixed, without the need to adjust information retrieval according to environmental changes, making it difficult to touch the core of the dynamic processing of ecological working memory. Therefore, its intervention effect is relatively weak.

In addition, numerous studies have confirmed that motor skills involving complex environments and complex movement structures imply higher complex cognitive demands and yield greater benefits for promoting inhibitory control. Overall, open motor skills present higher cognitive challenges and can facilitate the development of inhibitory control and working memory. Formenti et al. (2021) found that open motor skill exercise can promote the development of inhibitory control and motor skills. Takahashi and Grove (2023) used functional near-infrared spectroscopy (fNIRS) to compare the effects of badminton and running on inhibitory control and brain activation. They found that badminton exercise improved performance on the Stroop task without increasing brain activation, indicating that open-skill exercise can induce the optimization of neural efficiency. Similar results were further demonstrated in a study by Jiang et al. (2025).

However, this study has not yet found a more positive intervention effect of open-skill exercise on ecological cognitive flexibility. Compared with ecological inhibitory control and ecological working memory, ecological cognitive flexibility is relatively complex—it is a more sophisticated executive function structure built on the foundation of inhibitory control and working memory. Therefore, this complexity directly results in the need for longer intervention duration for changes at the neural and behavioral levels to become apparent. A systematic review conducted by Shi et al. (2022) revealed that exercise interventions exerted a significant effect on inhibitory control and working memory, while the same intervention duration yielded no notable impact on cognitive flexibility. This study further supports the viewpoint of the present research. Given that the intervention period of this study was 8 weeks, it may have been insufficient to induce an improvement in ecological cognitive flexibility.

### Open-skill exercise has a more prominent intervention effect on the mobile phone dependence of adolescents

The results of this study indicate that open-skill exercise is more effective in reducing phone dependence among adolescents. These findings are consistent with the research results of Liu et al. (2022). Their study (Liu et al., 2022) explored the intervention effects of 10 weeks of open-skill exercise (basketball group) and closed-skill exercise (Baduanjin group) on college students' phone dependence. It was found that in certain dimensions of phone dependence, the intervention effect of open-skill exercise was significantly better than that of closed-skill exercise.

The dependence of adolescents on mobile phones largely stems from the strong capture of their attention by the “high-stimulation, immediate feedback” environment created by mobile phones (Christodoulou and Roussos, 2025). The social interaction, dynamic contexts, and complex challenging task elements inherent in open skills can attract adolescents' attention and reduce their mobile phone dependence (Lepp et al., 2013; Zhu et al., 2020). In addition, such exercises require continuous attention to the environment and anticipation of opponents' movements, which consume a great deal of cognitive resources, reduces the allocation of attention to mobile phone information, and weakens the impulse to mindlessly scroll through mobile phones (Zhu et al., 2020). During open skill exercise, adolescents work closely with their teammates to pursue common goals, gaining a sense of social satisfaction and accomplishment in real life, thereby reducing the need to seek social comfort and virtual satisfaction through mobile phones (De-Sola Gutiérrez et al., 2016; Park, 2005).

In addition, the continuous improvement of executive functions during exercise enables adolescents to resist the urge to use mobile phones frequently, which also helps reduce their level of mobile phone dependence (Li et al., 2025). This study further confirms the potential mediating role of ecological executive functions in the intervention of mobile phone dependence through open skill exercise, and its explanation will be discussed in subsequent sections. However, due to the relatively limited social interactions in closed skill training and its relatively limited beneficial effects on attention and executive functions, its effectiveness in reducing the degree of mobile phone dependence is not as good as that of open skill training.

### The mediating role of ecological executive functions in the intervention of mobile phone dependence through open skill exercise

This study confirms that there is a significant negative correlation between ecological executive functions and mobile phone dependence, particularly evident in the dimensions of inhibitory control and working memory. Further mediating effect analysis reveals that ecological inhibitory control and ecological working memory play important mediating roles in the intervention of adolescents' mobile phone dependence through open skill exercise. Specifically, open skill exercise can not only directly reduce adolescents' mobile phone dependence, but also first enhance ecological inhibitory control and ecological working memory, and then indirectly reduce mobile phone dependence through these two abilities. These two factors serve as the key “bridges” connecting open skill exercise and the reduction of mobile phone dependence.

Firstly, open-skill exercise can effectively enhance adolescents' ecological inhibitory control and working memory. The core characteristics of open-skill exercise include high levels of social interaction, dynamic changes in the environment, and the need for collaborative completion of task objectives. These features precisely provide a “natural training ground” for ecological inhibitory control and working memory. This point has been discussed in detail and will not be elaborated on further here. Secondly, ecological inhibitory control and ecological working memory can inversely “constrain” mobile phone dependence. Well-developed ecological executive functions help adolescents better manage their behaviors and time, inhibit the impulse for excessive mobile phone use, flexibly adjust their lifestyles, and reduce reliance on mobile phones (Arns et al., 2007; Warsaw et al., 2021). Furthermore, adolescents with stronger inhibitory control can adhere to the usage rules they set when facing the temptation of mobile phones, thereby avoiding addiction. Those with more robust ecological working memory are able to focus on real-life goals and reduce the need for immediate stimulation from mobile phones. Ultimately, the degree of mobile phone dependence decreases as ecological executive functions improve.

### The innovations and practical value of this study

The innovations of this study are mainly reflected in three core dimensions: research perspective, assessment tool, and mechanism verification. From the research perspective, the study systematically compares the intervention effects of two types of exercises (open and closed motor skills) in the community context, filling the research gap in the field of sports types and adolescent behavioral addiction intervention. In terms of the assessment tool, it abandons the traditional laboratory computer-based tasks that are disconnected from real-life scenarios, and instead adopts a questionnaire designed around adolescents' daily scenarios, focusing on the ability to apply executive function in real life, which enhances the external validity of the research results and their practical guiding significance. In terms of mechanism verification, the study innovatively constructs and verifies a mediating effect model, clarifies the partial mediating role of ecological executive function between open-skill sports and adolescents' mobile phone dependence, and enriches the theoretical system in the field of “exercise-cognition-behavioral addiction”.

In terms of practical application, this study provides a practical case for communities to carry out mobile phone dependence intervention among adolescents. Communities can rely on existing sports venues to set up “open skill sports camps” for adolescents with mobile phone dependence tendencies, and strengthen adolescents' executive functions through dynamic tasks such as team offense and defense, and tactical cooperation, thereby reducing their level of mobile phone dependence. Regarding promotion suggestions, this study recommends that local governments incorporate the construction of open skill sports facilities for adolescents into community planning. When building new communities or renovating old communities, priority should be given to constructing venues such as basketball courts and small football fields, along with basic sports equipment. Meanwhile, professional coaches should be introduced through government-purchased services to provide teacher support for community sports interventions and reduce operational costs.

### Limitations and future research prospects of this study

Although this study initially confirms that community-based open-skill exercises are more conducive to improving adolescents' ecological executive function and reducing their mobile phone dependence, it still has limitations in several aspects. First, the sample size is small and its regional representativeness is limited. A small sample size may not only restrict the overall statistical power and reduce the statistical stability of the research results, but also affect the statistical power of the mediating effect test. Second, the intervention duration is relatively short. This study set the intervention duration at 8 weeks; although significant intervention effects of exercise on ecological executive function and mobile phone dependence were observed, it is impossible to verify the long-term sustainability of these effects, nor to rule out the possibility of a rebound in mobile phone dependence after the intervention is terminated. Third, there are deficiencies in the blinding design. Due to the significant differences in intervention formats between open-skill and closed-skill exercises, and the fact that participants can easily recognize the differences between groups, only outcome assessor blinding was adopted in this study. Fourth, there is a lack of long-term follow-up, making it impossible to track the duration and dynamic changes of the intervention effects.

Based on the above limitations, this study puts forward the following prospects for future research. First, it is recommended to expand the sample size and include more adolescent participants to improve the overall statistical power and the reliability of results; in particular, for the mediating effect test, a larger sample size is needed to enhance statistical efficiency. Second, it is suggested to extend the intervention duration and add follow-up assessments at multiple time points after the intervention ends, so as to track the changing trends of participants' ecological executive function and mobile phone dependence, verify the sustainability of the intervention effects, and provide a scientific basis for formulating long-term intervention strategies. Third, future studies are advised to optimize the blinding design. For example, more concealed intervention protocols can be designed to achieve blinding for both participants and intervention implementers as much as possible, thereby reducing the impact of subjective biases on the research results.

## Conclusions

Taking the community as the intervention setting, this study explored the intervention effects of open-skill and closed-skill exercises on adolescents' ecological executive function and mobile phone dependence, and verified the mediating role of ecological executive function. The results showed that open-skill exercises had a more significant intervention effect on adolescents' ecological executive function and a more prominent effect in reducing adolescents' mobile phone dependence. In addition, this study further found that ecological executive function played a partial mediating role between open-skill exercises and mobile phone dependence. This conclusion not only enriches the research evidence in the field of “exercise type—cognitive function—behavioral addiction”, but also provides a scientific basis for communities to formulate targeted intervention programs for adolescent mobile phone dependence. Specifically, promoting open-skill exercises can help address the public health and social issue of excessive mobile phone dependence among adolescents.

## Data Availability

The raw data supporting the conclusions of this article will be made available by the authors, without undue reservation.
